# Challenges and Opportunities of Real-World Data: Statistical Analysis Plan for the Optimise:MS Multicenter Prospective Cohort Pharmacovigilance Study

**DOI:** 10.3389/fneur.2022.799531

**Published:** 2022-03-28

**Authors:** Ed Waddingham, Aleisha Miller, Ruth Dobson, Paul M. Matthews

**Affiliations:** ^1^Department of Brain Sciences and Dementia Research Institute, Imperial College London, Hammersmith Campus, London, United Kingdom; ^2^Preventive Neurology Unit, Wolfson Institute of Preventive Medicine, Queen Mary University of London, London, United Kingdom

**Keywords:** real-world data, cohort study, signal detection, multiple sclerosis, pharmacovigilance, statistical analysis plan

## Abstract

**Introduction:**

Optimise:MS is an observational pharmacovigilance study aimed at characterizing the safety profile of disease-modifying therapies (DMTs) for multiple sclerosis (MS) in a real world population. The study will categorize and quantify the occurrence of serious adverse events (SAEs) in a cohort of MS patients recruited from clinical sites around the UK. The study was motivated particularly by a need to establish the safety profile of newer DMTs, but will also gather data on outcomes among treatment-eligible but untreated patients and those receiving established DMTs (interferons and glatiramer acetate). It will also explore the impact of treatment switching.

**Methods:**

Causal pathway confounding between treatment selection and outcomes, together with the variety and complexity of treatment and disease patterns observed among MS patients in the real world, present statistical challenges to be addressed in the analysis plan. We developed an approach for analysis of the Optimise:MS data that will include disproportionality-based signal detection methods adapted to the longitudinal structure of the data and a longitudinal time-series analysis of a cohort of participants receiving second-generation DMT for the first time. The time-series analyses will use a number of exposure definitions in order to identify temporal patterns, carryover effects and interactions with prior treatments. Time-dependent confounding will be allowed for *via* inverse-probability-of-treatment weighting (IPTW). Additional analyses will examine rates and outcomes of pregnancies and explore interactions of these with treatment type and duration.

**Results:**

To date 14 hospitals have joined the study and over 2,000 participants have been recruited. A statistical analysis plan has been developed and is described here.

**Conclusion:**

Optimise:MS is expected to be a rich source of data on the outcomes of DMTs in real-world conditions over several years of follow-up in an inclusive sample of UK MS patients. Analysis is complicated by the influence of confounding factors including complex treatment histories and a highly variable disease course, but the statistical analysis plan includes measures to mitigate the biases such factors can introduce. It will enable us to address key questions that are beyond the reach of randomized controlled trials.

## Introduction

Optimise:MS is a prospective observational cohort study lasting at least 7 years (with the possibility of extension depending on funding), focused on evaluating the safety profile of MS DMTs in the real-world setting. A sample size of around 4,000 multiple sclerosis (MS) patients is anticipated, to be recruited from several sites (MS treatment centers) around the UK. This sample size is based on the recruitment level that is expected to be achievable in practice, rather than on considerations relating to statistical power. The study is open to all MS patients [as defined by the 2017 McDonald criteria (1)], of any MS subtype, attending a participating site and eligible for treatment based on current UK guidelines (2), regardless of their actual treatment history. The study has been recruiting since May 2019, and as of 2022 remains open to new recruits. The length of the recruitment window, coupled with the introduction of remote consenting, should ensure that the sample is not heavily skewed toward those attending clinics most frequently. Details of the study design and protocol have already been published (3). The study is academically initiated and led, but is guided by a public-private partnership between academic clinical investigators and pharmaceutical companies with marketing authorisations for DMTs.

Subjects taking second-generation DMTs will be the main focus of investigation, and controls will include those eligible but not receiving treatment and those receiving first-generation DMTs (see Table 1 for a current list of first- and second-generation DMTs; any new DMTs becoming available for use by patients in the UK during the course of the study will be classed as second-generation).

**Table 1:** Classification of DMTs in the Optimise:MS study.

**Drug**	**Product name(s)**	**Mode and frequency of delivery**
**First-generation DMTs**		
Glatiramer acetate	Brabio, Copaxone	Subcutaneous, 3–7 × weekly
Interferon beta-1a	Avonex	Intramuscular, weekly
Interferon beta-1a	Rebif	Subcutaneous, 3 × weekly
Pegylated interferon beta-1a	Plegridy	Subcutaneous or intramuscular, every 2 weeks
Interferon beta-1b	Betaferon, Extavia	Subcutaneous, every 2 days
**Second-generation DMTs**		
Alemtuzumab	Lemtrada	Intravenous infusion, 5 consecutive days followed by 3 consecutive days 1 year later
Cladribine	Mavenclad	Oral, up to 5 consecutive days per month for 2 months, repeated 1 year later
Daclizumab	Zinbryta	Subcutaneous, monthly
Dimethyl fumarate	Tecfidera	Oral, 2 × daily
Fingolimod	Gilenya	Oral, daily
Natalizumab	Tysabri	Intravenous infusion, monthly
Ocrelizumab	Ocrevus	Intravenous infusion, 2 × yearly
Ofatumumab	Kesimpta	Subcutaneous, monthly
Rituximab	Mabthera, Truxima	Intravenous infusion, up to 2 × yearly
Siponimod	Mayzent	Oral, daily
Teriflunomide	Aubagio	Oral, daily

The primary objective of the study is to establish the incidence of serious adverse events (SAEs) among MS patients receiving any second-generation DMT, and compare it with that observed in untreated but treatment-eligible patients and those receiving first-generation DMT.

Secondary objectives are:

- to measure and compare SAE rates for individual DMTs;
- to assess associations between second-generation DMT therapy and incidence of lymphopenia;
- to assess associations between second-generation DMT therapy and moderately and severely abnormal liver function, as indicated by blood tests for alanine transaminase or aspartate transaminase;
- to assess the impact of sequential DMT therapy on the incidence of SAEs;
- to assess the relative efficacy of DMT classes with regard to suppression of relapses, disability progression and new lesion formation on MRI;
- to measure the frequencies of pregnancies and their outcomes.

SAEs are defined as adverse events resulting in death, persistent or significant disability/incapacity, or hospitalization (or extension of a hospital stay for an inpatient). These are classified according to the following categories: Opportunistic infections, infections requiring hospitalization, MS relapses, deaths, COVID-19 infections, other SAEs deemed to be related to treatment (e.g., malignancies), and other SAEs.

## Methods and Analysis

### Study Sites, Data Entry, and Storage

Participants are recruited at participating MS clinics at hospitals around the UK. Currently there are 14 participating hospital sites and over 2,000 individuals have been enrolled in the study.

At each site, study data is entered onto a local secure database held on a dedicated PC. These machines connect securely to the Optimise:MS server (hosted by the Data Science Institute at Imperial College London) and automatically upload (“push”) the data to the central database at regular intervals. Regular quality checks on the data central data are performed centrally through monitoring data completeness, internal consistency, concordance with expected ranges, and harmonization of units; queries are fed back to the site staff for resolution.

Participants' data is managed in line with the requirements of the General Data Protection Regulation, Imperial College London's policies and the study's own Standard Operating Procedures. Personally identifiable data is kept to a minimum; names and contact details are accessible only by local site staff and are not stored on the central study database.

### Longitudinal Cohort Structure and Outcome Assessment

MS patients may join the study if they are eligible for treatment with DMT, regardless of whether or not they actually receive DMT. Upon enrolment the patient's basic demographic and clinical data (including their MS diagnosis and any comorbidities) are entered onto the study database by site staff. Retrospective data is also collected at enrolment, including disability assessments and relapses, lab test results, a full history of DMT use, and any past serious infections or malignancies.

Whenever a participant attends a clinic visit while under observation in the study, the database is updated with the reason for the visit, date of the visit, and details of any other changes in the participant's data (such as disease progression, new comorbidities, any treatment changes, SAEs, test results, or MRI scan results) since the previous visit. Exact dates for all such events are recorded whenever possible. No additional clinic visits or procedures are required as part of the study. Participants are under observation from their enrolment visit until they withdraw consent, leave a participating clinic, die, or until the end of the study, whichever is the earliest.

SAEs (including MS relapses), pregnancies and their outcomes, and any new/enlarging lesions revealed by clinically indicated interval MRI are recorded on the Optimise database by local site staff accessing medical records. Disability is assessed by local clinical staff using the Expanded Disability Status Scale (EDSS) (4) and the total score is recorded on the database; a disability progression outcome is defined as an EDSS measurement scoring at least 1 point higher than the most recent measurement at or after baseline. Laboratory test results (e.g., blood cell and liver enzyme counts) also are recorded on the database. Abnormal liver function is assessed using blood alanine aminotransferase (ALT) or aspartate aminotransferase (AST) levels. For each, moderate and severe elevation are, respectively, defined as exceeding 2.5 × and 5 ×, respectively, of the upper limit of the normal ranges established by Imperial North West London Pathology. Lymphopenia is defined based on absolute lymphocyte count (ALC) according to the following grades:

- Grade 1: Lower limit of normal range ≥ ALC ≥ 800/mm^3^
- Grade 2: 800/mm^3^ ≥ ALC ≥ 500/mm^3^
- Grade 3: 500/mm^3^ > ALC ≥ 200/mm^3^
- Grade 4: 200/mm^3^ > ALC

### Statistical Principles

Due to selection effects, MS patients receiving different treatments are likely to have different underlying characteristics and to experience outcomes at different rates even before allowing for the effects of treatment. Thus, confounding is expected between treatment selection and outcomes, leading to biased treatment effect estimates. Confounding variables may include demographics, disease and treatment history, and time variables representing period and cohort effects (5).

Controlling for the effects of confounders can be particularly difficult in longitudinal studies where past treatment exposures and covariates may influence future exposures and/or covariates as well as future outcomes. This is known as time-varying causal pathway confounding, and the bias it introduces may not be adequately controlled by the standard multivariate covariate adjustment approach (6–8). This type of confounding is expected to occur in the Optimise:MS cohort, given the nature of MS as a chronic progressive disease and the factors that are suspected to influence treatment decisions. Methods for controlling confounders have been chosen to mitigate this problem (further details below).

The statistical analyses fall into three classes: cohort analyses, signal detection analyses, and pregnancy analyses. These are described under the headings below.

### Cohort Analyses

A “new user” cohort of those study subjects who have never received second-generation DMT prior to study enrolment will be the subject of longitudinal analyses. These will examine the effects of DMTs on relapse, disability progression, abnormal liver function, lymphopenia, new lesion formation and SAE rates. The temporal relationship between exposures and outcomes will also be explored.

**Figure 1:**
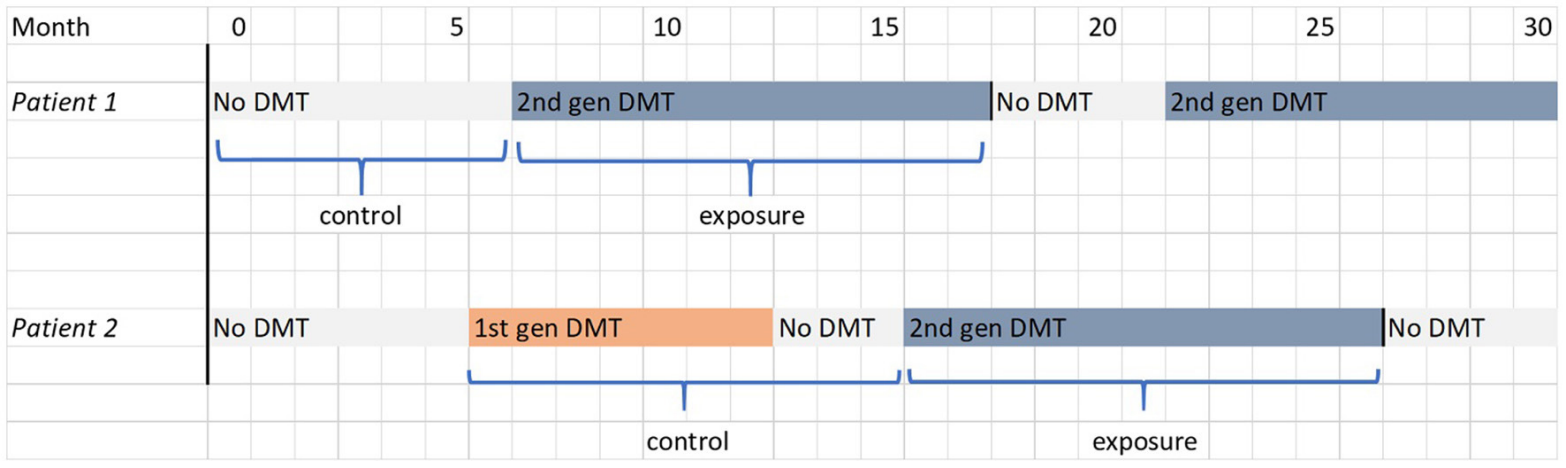
FigIllustrations of the determination of exposure and control periods in the primary cohort analysis for two hypothetical patients, one in each stratum. The filled blocks represent the treatment received by the patient; the labels below indicate the periods of follow-up that contribute to the analysis.

The primary cohort analysis aims to investigate the effectiveness and safety of DMTs using a relatively simple model. Participants will be separated into two strata according to whether or not they have ever received first-generation DMT prior to second-generation DMT initiation (or prior to the end of follow-up, if second-generation DMT is never initiated). Within each stratum, outcomes occurring while exposed to second-generation DMT will be compared to outcomes occurring while unexposed. Follow-up is censored upon cessation of second-generation DMT. Subjects who commence second-generation DMT while under observation will contribute an initial unexposed episode and a subsequent exposed episode of follow-up time to the analysis, as illustrated for two hypothetical patients in Figure 1.

To control for confounding in the primary analysis, propensity score weighting will be used; each exposure episode will be weighted in inverse proportion to the estimated propensity (probability) of the observed treatment exposure. The propensity score is based on time-varying covariates measured at the start of the exposure episode (7). The effect of the weighting is to construct a pseudo-population which is effectively “randomized” in the sense that the covariates at the start of exposure episodes are balanced across exposure categories. The propensities are estimated using a pooled logistic regression model.

**Figure 2:**
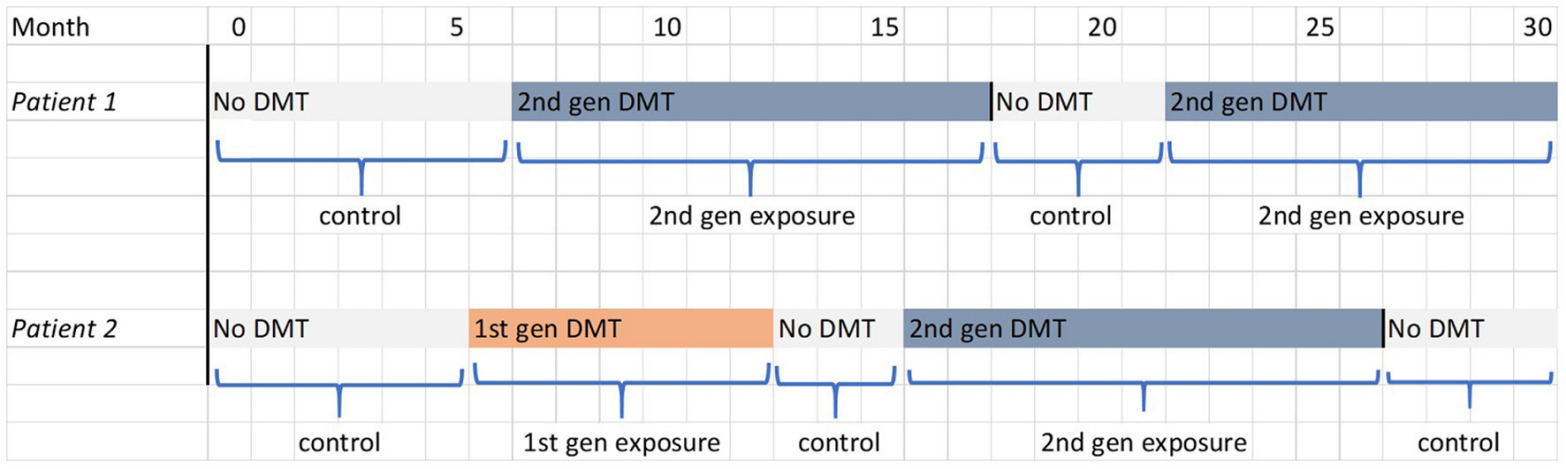
Illustration of the determination of exposure and control periods in the secondary and tertiary cohort analysis for the two hypothetical patients shown in Figure 1. The filled blocks represent the treatment being received by the patient; the labels below indicate the periods of follow-up that contribute to the analysis.

The secondary cohort analyses are aimed at exploring the temporal relationship between DMT use and outcomes, including whether the effects of DMTs persist after treatment cessation/switch. Follow-up is not censored upon cessation of second-generation DMT; instead, participants can contribute multiple periods of exposure to the analysis as they move between treatment classes. This is illustrated in Figure 2 for the two hypothetical patients described in Figure 1. The secondary analyses thus make use of all observed data for the new user cohort and, owing to the more complex longitudinal exposure patterns involved, observations will be weighted using time-varying inverse probability weights (IPTW) to estimate a marginal structural model (MSM) (9). This is similar to the propensity score method described above, but the weights are updated at regular (6-month) intervals based on the latest covariate values and reflect the probability of observing the participant's full treatment history up until that timepoint (6). For details of how these probabilities are modeled, and the formulae for the weights, see the Supplementary Material. This method aims to create a dynamically weighted pseudo-population that is longitudinally balanced, i.e., with covariates equally balanced across all possible treatment histories at every 6-month timepoint. This construction relies on an assumption that the probabilities lie strictly between 0 and 1 for each possible level of the covariates (the positivity assumption). Provided that this condition is met and all confounders are measured at sufficiently frequent intervals, this method can fully control for time-varying causal pathway confounding and generate unbiased estimates of the marginal treatment effects. A three-category treatment variable will be used (no treatment, first-generation DMT or second-generation DMT) instead of the stratified approach of the primary analysis. Parallel analyses will use different exposure models to examine the temporal patterns of treatment effects:

(a) Outcomes associated with current treatment class (categorical exposure variable).
(b) Outcomes associated with current treatment class plus carryover effect of any other treatment class in the past 6 months (categorical exposure variables).
(c) Outcomes associated with cumulative exposures (continuous exposure variable for each treatment category).
(d) Outcomes associated with time-weighted cumulative exposure, i.e., historic exposures downweighted relative to recent exposures (continuous exposure variable for each treatment category).

The tertiary cohort analysis extends exposure model (b) to examine whether there is an interaction effect associated with treatment switching, i.e., whether the carryover effect of previous treatment is dependent on current treatment exposure.

Further cohort analyses will examine the effects of second-generation DMTs individually rather than as a collective treatment class. The principle analysis method for all cohort analyses will be time-varying Cox proportional hazards regression (10).

### Signal Detection Analyses

The signal detection analyses will examine whether the rate of SAEs (excluding MS relapses) occurring for any individual DMT is disproportionate to the overall rate of SAEs in the study sample. SAEs will be analyzed according to their classification sin the Optimise database as:

- Infections
- Opportunistic Infections
- Malignancies and other SAEs likely related to treatment
- Deaths (all causes)
- Covid-19
- Other SAEs

Infections, opportunistic infections and Covid-19 will be further analyzed according to the subtypes recorded on the database, currently including the following categories:

- *Infections*: urinary tract infections, bronchitis, sinusitis, gastroenteritis, thinea, sepsis, bacterial, viral, abscess, other.
- *Opportunistic Infections*: progressive multifocal leukencephalopathy, herpes zoster, herpes simplex, varicella,viral hepatitis, listeria, mycosis, abscess, other.
- *Covid-19*: suspected, confirmed by test, hospitalized, ventilated.

Classifications based on MedDRA codings or free-text descriptions may also be used.

Patient-months will be assigned to treatments according to three different definitions of exposure:

- Exposure within the month of interest or the previous month.
- Exposure within the preceding 6 months.
- Exposure at any prior time in the patient's treatment history.

Only incident events (i.e., the first recorded occurrence in a given study participant) will be analyzed; follow-up is censored upon occurrence of the event of interest.

A minimum report criterion is also imposed in order to avoid statistical noise in the disproportionality statistics when event counts are too low. For a signal to be triggered, an event must be reported in at least 3 study participants for second-generation DMTs and 5 participants for first-generation DMTs. The higher threshold in the latter case results in fewer false positives and more precise risk estimates, but with reduced sensitivity (11), reflecting the fact that the safety profile of first-generation DMTs is relatively well understood and early detection of signals is less of a priority than for the newer treatments.

#### Signal Detection Methodologies/Measures

The key disproportionality methods used in this study, the Reporting Odds Ratio and Bayesian Confidence Propagation Neural Network, were originally developed in the context of spontaneous report databases. In this original context the methods would be used to evaluate whether an event is cited more frequently in AE reports for the treatment of interest than in reports for other treatments.

Longitudinal cohort data also covers periods when no adverse events occur, which provides additional information regarding the relative frequencies of exposures and outcomes. When applying the disproportionality approach in the longitudinal setting it is appropriate to make use of this additional data by altering the methods so that they do not simply count AE reports occurring on treatments, but also take into account periods with no exposure and/or no events (12). This is achieved by treating each patient-month of follow-up as a unit of observation and evaluating whether events occur more frequently during patient-months exposed to the treatment of interest than during all other patient-months. The methods are described under the headings below in accordance with this longitudinal formulation.

##### Simple Disproportionality Measures

The reporting odds ratio (ROR) (13) compares the odds of an adverse event occurring during exposed patient-months to the odds of occurrence during unexposed patient-months. For a given drug-event combination the ROR is calculated as follows:


$$\begin{array}{l} ROR=\frac{n_{11}n_{00}}{n_{01}n_{10}} \end{array}$$

Where *n*_00_ = number of patient-months without exposure to drug or occurrence of event

*n*_01_ = number of patient-months without exposure to drug but with occurrence of event

*n*_10_ = number of patient-months with exposure to drug but without occurrence of event

*n*_11_ = number of patient-months with exposure to drug and occurrence of event

Another simple disproportionality measure is the proportional reporting ratio (PRR), which is calculated not as an odds ratio, but rather a relative risk in exposed vs. unexposed months:


$$\begin{array}{l} PRR=\frac{n_{11}n_{0.}}{n_{01}n_{1.}} \end{array}$$

where the dot symbol “·” indicates summation over the index values 0 and 1 (14). A third measure is the relative reporting ratio (RRR), a relative risk in exposed vs. all months:


$$\begin{array}{l} RRR=\frac{n_{11}n_{..}}{n_{.1}n_{1.}} \end{array}$$

In practice the PRR, RRR and ROR give near-identical results when used for signal detection (12, 15).

The incidence rate ratio (IRR) is a standard relative measure of incidence in epidemiology and medical statistics, often estimated by Poisson regression. It is calculated as the incidence of an event among treated participants divided by its incidence among untreated participants, where the incidence is the number of events divided by the total amount of follow-up time. It can easily be seen that the IRR is equivalent to the longitudinal formulation of the PRR described above. This observation allows us to calculate a confounder-controlled estimate of the PRR *via* weighted Poisson regression, using the marginal structural approach described under “Cohort Analyses” above. Indeed, the same weighted PRR estimate can be obtained by directly substituting weighted equivalents of *n*_01_, *n*_11_, *n*_00_, and *n*_10_ in the formula above (for details see the Supplementary Material). The latter approach can be extended to calculate a weighted version of the RRR, which will be used in the “weighted analysis pathway” (see Section Signal Generation Procedure below).

##### Shrinkage (Bayesian Confidence Propagation Neural Network)

Owing to the discrete nature of count data, simple disproportionality measures are very unstable when event rates are low. Chance occurrences of a rare event can easily generate spurious false positive signals.

The Bayesian Confidence Propagation Neural Network (BCPNN) method (16) is designed to reduce the rate of false positives by using a Bayesian model to express the joint distribution of the probabilities of drug exposure and event occurrence, with conjugate beta priors that favor an independent relationship (i.e., no association between drug and event). This achieves a “shrinkage” effect that pulls the disproportionality estimates back toward the null when event counts are low.

The model's key measure of disproportionality is the Information Component, which is the base-2 logarithm of the RRR. A posterior estimate of the False Discovery Rate (FDR) for each signal, i.e., the probability of no association between drug and event, can also be obtained (17).

##### Controlling for Protopathic Bias (LEOPARD)

Signal detection methods are often prone to generating false positives due to protopathic bias, which occurs if an event is mistakenly ascribed to initiation of a new treatment when both shared a common cause such as an underlying disease exacerbation (18). LEOPARD is a signal filtering method aimed at eliminating this bias. The method works by examining the rate of treatment initiations before and after adverse event incidence; protopathic bias is inferred if treatment follows the event more often than it precedes it (15). To address this, we will employ a one-sided binomial test of the distribution of treatment initiation events, with the null hypothesis that treatment initiation is equally likely before an AE as after it, and the alternative hypothesis that the probability is higher after the AE. This test will be carried out at the 50% significance level (19); signals where the null hypothesis is rejected will be discarded.

##### Signal Generation Procedure

**Figure 3:**
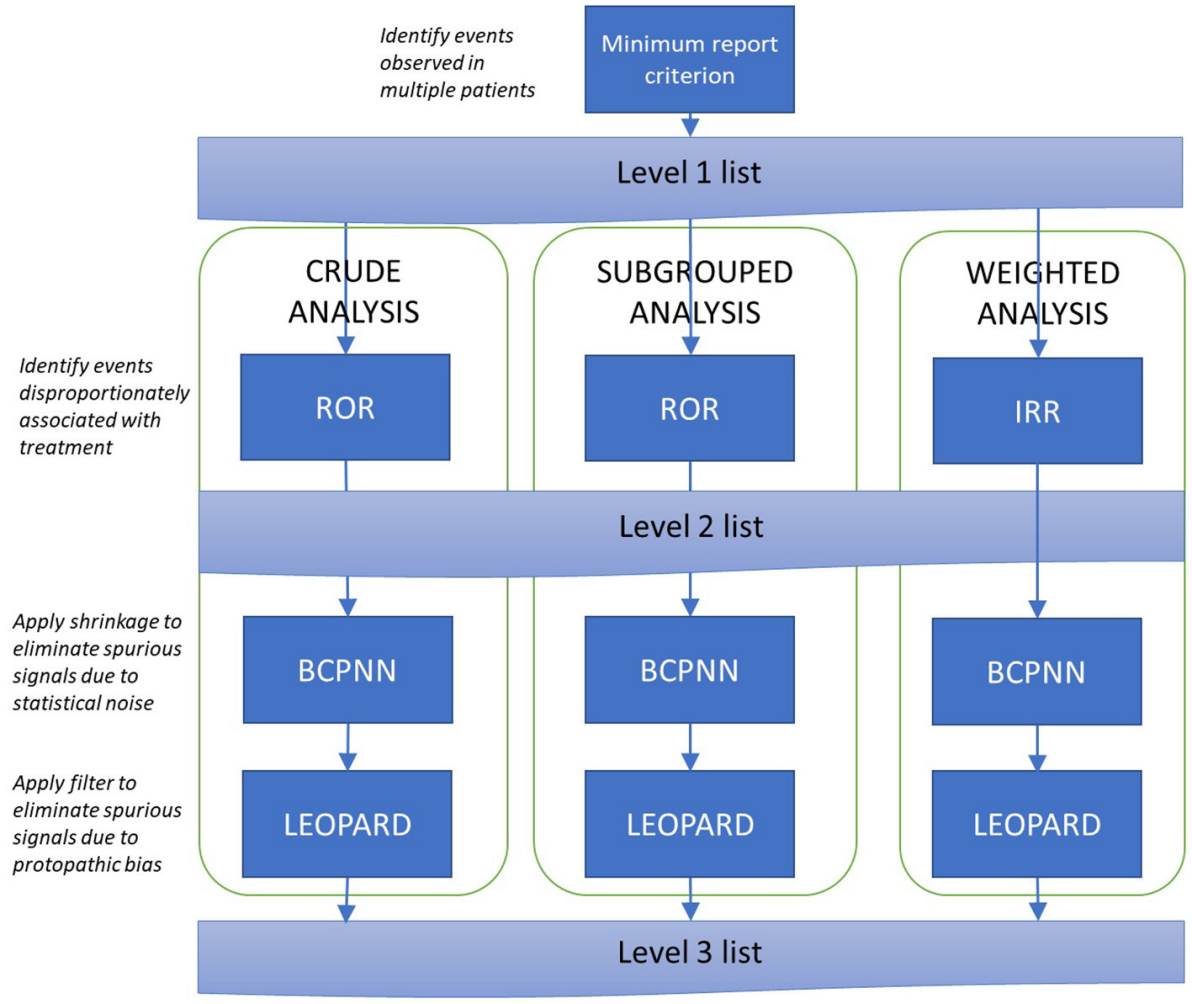
Signal generation procedure. ROR, Reporting Odds Ratio; IRR, Incidence Rate Ratio; BCPNN, Bayesian Confidence Propagation Neural Network.

For each treatment of interest and exposure definition, the analysis will follow the process set out in Figure 3. As the first step in the analysis, a list of events fulfilling the minimum report criterion is generated (the Level 1 list). Thereafter, three parallel analysis pathways are used: a crude (unadjusted) disproportionality analysis, and two analyses aimed at controlling for potential confounding covariates: a subgrouped analysis and a weighted analysis (IPTW).

Within each pathway, a Level 2 list is produced containing all signals identified by the Reporting Odds Ratio or, equivalently, the incidence rate ratio. Signals are triggered when the lower 95% confidence bound for the disproportionality measure exceeds 1 [for the subgrouped analysis, this must be observed in at least one subgroup; this approach has been reported to provide better performance than using a pooled odds ratio (11)].

The Level 2 list is expected to contain some false positives due to (i) volatility of disproportionality measures associated with low event counts, and (ii) protopathic bias. The Level 3 list tackles these problems by (i) applying Bayesian shrinkage to pull disproportionality estimates back toward the null (the Bayesian Confidence Propagation Neural Network Method) and (ii) verifying that prescriptions tend to precede rather than follow events (the LEOPARD filter). Signals with an FDR estimate below 5% which are not rejected by the LEOPARD filter will be included on the Level 3 list. In the subgrouped- analysis, these conditions must be achieved in at least one sub-group; in the weighted analysis, the BCPNN calculations are based on the weighted event counts described in the Supplementary Material.

Pooled lists at levels 2 and 3 will be produced in which signals will be ranked according to the number of pathways in which the signal was observed and the associated disproportionality statistics (level 2) or estimated false discovery rates (level 3) (17).

Sensitivity analyses may explore the use of alternative decision rules, such as varying the minimum report or FDR thresholds, and alternative methodologies, such as replacing BCPNN with the Gamma-Poisson Shrinker (15, 20) or Information Component Temporal Pattern Discovery (21).

After drug-event signals have been identified, the data will be further examined for evidence of drug-drug-event signals, i.e., adverse events associated with treatment interactions. These analyses will also proceed using the procedure set out in Figure 3, with different exposure definitions and background rates depending on the context (these are set out in the full Statistical Analysis Plan).

An additional pediatric signal detection analysis be carried out in participants under 18 years old. For this purpose the threshold for the minimum report criterion will be reduced to 2 cases, and only the crude analysis pathway will be used.

### Pregnancy Analyses

The average rate of pregnancy per person-year of follow-up will be estimated, both among all females aged 18–50 in the study population and according to DMT class and specific DMT being received at the date of conception.

Multinomial or binomial logistic regression will be used to estimate the effect of the treatment received at conception on the eventual outcome of pregnancy.

### Planned Interim Analyses

The study is in a position to reveal previously unobserved adverse drug reactions, particularly in connection with the more novel second-generation DMTs. To facilitate timely detection of such signals, a simplified set of analyses will be performed on an annual basis while data is being accrued. These will consist of the signal detection analyses (crude analysis pathway and single-drug-event associations only), and simple (constant-hazard) unadjusted Poisson regressions of the occurrence of any SAE according to current treatment received.

## Discussion

Optimise:MS is being carried out in a routine sub-specialty referral care setting, and will thus provide “real-world” data on outcomes occurring under the sort of treatment and clinical monitoring regimes that patients typically experience, rather than the idealized conditions of a randomized controlled trial (RCT) (22). The study participants should be more representative of the general population of MS patients in the UK than would be the case in a typical RCT, since the inclusion criteria are less restrictive and the study does not burden the participants with additional procedures or impose any new treatment regimes. This also facilitates recruitment, and over a long period of follow-up, despite the lack of additional investigations or procedures, enables a comprehensive set of clinical data to be gathered. The use of electronic consent forms and remote/virtual clinic visits has also helped in this regard, particularly during the COVID-19 pandemic.

The sample size and length of follow-up thus exceed most RCTs and, together with the detailed data gathered on participants' DMT and disease histories, will enable the estimation of washout, switching and subgroup effects that often lie beyond the scope and capabilities of trials.

Of course, observational studies have well-known drawbacks compared to RCTs—chiefly the absence of randomization, which leaves treatment selection potentially subject to the influence of prognostic factors and therefore vulnerable to confounding with outcomes. The likely existence of time-varying causal pathway confounding in the MS context makes this problem particularly challenging to address analytically, but the marginal structural modeling approach (IPTW) has shown that it has the capability to produce unbiased estimates—at least under ideal conditions when positivity is satisfied, probability models are specified correctly and there are few extreme weights (8, 23, 24). The comprehensive longitudinal data collection in Optimise should facilitate MSM estimation, which will be particularly important for the secondary cohort analyses investigating the effect of longitudinal treatment trajectories. The estimation of probability weights in itself may provide useful insight into the prevalence of DMT use in particular subgroups, and other factors influencing treatment decisions.

We have also specified a simpler cross-sectional propensity-score weighting approach, as this improves the chances of positivity and reduces the potential for extreme weights. Although this model may not fully control for the influence of prior treatment history on outcomes, this is less likely to be a major concern in the primary cohort analysis since exposure histories are relatively simple (Figure 1) compared to the more complex exposure histories in the secondary analysis (Figure 2).

The use of weighted event counts in the disproportionality-based signal detection methods is, to our knowledge, novel, but is well-founded (see the Supplementary Material). This is the only method we are aware of that can control for time-varying causal pathway confounding when using disproportionality methods such as the ROR, BCPNN, or GPS. However, it can only be used when these methods are applied to longitudinal cohort data, rather than to the spontaneous report data for which such methods were originally developed. Linking cases to their treatment histories, and hence examining drug-drug-event signals involving washout effects of prior treatments, is also more straightforward in the longitudinal setting. These considerations favor the Optimise:MS cohort-based design for future signal detection databases. Another reason, of course, is the additional data gained from periods with no treatment exposure or adverse events, which may improve the performance of disproportionality methods (15). Without this additional data, disproportionality analyses of spontaneous reports can unfairly penalize drugs with low overall AE rates if any one AE occurs more often than others (an example is shown in the Supplementary Material). Alongside the novel weighted analysis, a parallel subgrouped- analysis provides another means of controlling for confounders and is better established in signal detection (11, 20)—although this method may still be vulnerable to time-varying causal pathway confounding. since the subgroups are based on cross-sectional covariate values rather than full exposure and covariate histories.

A disadvantage of using the Optimise:MS study for signal detection purposes, as opposed to a spontaneous report registry, is the relatively small sample size. This exacerbates the known problem of volatility in disproportionality statistics when event counts are low—hence the importance of using a shrinkage methodology such as BCPNN. Protopathic bias presents another significant problem for pharmacovigilance in MS patients, as false signals may easily be generated by both the relapsing/remitting and progressive aspects of the disease, and the wide range of symptoms it can produce. Direct comparisons between safety profiles of different DMTs—in particular between first- and second-generation DMTs—may also be biased due to the fact that exposure and follow-up time are more limited for newer drugs, and so treatment effects that manifest over the longer term cannot be observed. Finally, the potential for differences in the intensity of follow up on different treatments to bias event detection is not specifically accounted for in the analysis. The impact of this varies greatly by outcome; for example, it would be expected to be greater for imaging measures of disease activity such as new or enlarging lesions than for SAEs. Although imaging results may also be affected by the use of different scanners, acquisition protocols and schedules, this is not expected to be strongly related to treatment.

In summary, Optimise:MS is observational, inclusive, and does not impose any fixed timelines on those taking part. Participants can be enrolled at any stage of their MS or treatment history; there is no unifying milestone marking for the start of follow-up, and no set course of treatment to be followed thereafter. This inclusivity makes recruitment easier, enhances data collection and may increase the population representativeness and generalizability of results, but it presents major challenges from a statistical perspective. We have tried to address these and realize opportunities arising from the design. Our approach to signal detection analyses will ensure a healthy mix of data from as wide a population as possible, although care has been needed to plan the analysis in a way that controls for treatment selection and protopathic bias. For longitudinal cohort analyses, the lack of fixed timelines for participants is a complicating factor, but also creates the potential for a wealth of useful data if handled appropriately. Our cohort analyses simplify the structure of the data by focusing on a sub-population of participants initiating second-generation DMT for the first time, as it is the safety profile of these drugs that is the primary outcome of interest. Further analytical choices have been made to either mitigate the confounding influence of variability in patient characteristics/histories (e.g., marginal structural modeling) or exploit this variability to gain additional insights (e.g., the analyses of washout/cumulative/switch effects).

## Author Contributions

EW developed the Statistical Analysis Plan with support and input from RD, AM, and PM and drafted the manuscript. RD and PM contributed to conception and design of the study and revisions of the manuscript. RD drafted the study protocol with contributions from PM. AM co-ordinated the activation and ongoing management of the study. All authors contributed to reviewing the manuscript and approved the submitted version.

## Funding

PM acknowledges generous personal and research support from the Edmond J. Safra Foundation and Lily Safra, an NIHR Senior Investigator Award, the UK Dementia Research Institute and the NIHR Biomedical Research Center at Imperial College London. The study was funded by a partnership comprising: Biogen IDEC Ltd. (Grant Reference WMCR_P76049, CrossRef Funder ID: 10.13039/100006314), Merck Serono Ltd., Feltham, UK, an affiliate of Merck KGaA, Darmstadt, Germany (Grant Reference WMCN_P74840, CrossRef Funder ID: 10.13039/100009945), and Celgene Ltd. (Bristol-Myers Squibb) (Grant ref WBCN_P79026, CrossRef Funder ID: 10.13039/100006436). The funding companies are represented on the study's Steering Committee but are not involved in day-to-day administration of the study, data collection or analysis. Manuscript publication fees are paid by Imperial College London.

## Conflict of Interest

The study received funding from Biogen IDEC Limited, Merck Serono Ltd. and Celgene Ltd. The statistical analysis plan and this manuscript were developed with input from the funders in a reviewing capacity. PM acknowledges consultancy fees from Novartis, Bristol Myers Squibb, Celgene and Biogen. He has received honoraria or speakers' honoraria from Novartis, Biogen and Roche and has received research or educational funds from Biogen, Novartis, GlaxoSmithKline, and Nodthera. RD works within the PNU, which was funded by Barts Charity. She receives grant support from the UK MS Society, BMA foundation, NIHR, MRC, NMSS, Horne Family Charitable Trust, Biogen and Merck. She has received honoraria for Advisory boards and/or educational activities from Biogen, Teva, Sanofi, Merck, Janssen, Novartis, and Roche. The remaining authors declare that the research was conducted in the absence of any commercial or financial relationships that could be construed as a potential conflict of interest.

## Publisher's Note

All claims expressed in this article are solely those of the authors and do not necessarily represent those of their affiliated organizations, or those of the publisher, the editors and the reviewers. Any product that may be evaluated in this article, or claim that may be made by its manufacturer, is not guaranteed or endorsed by the publisher.
