# Supplementary material for: Challenges and Opportunities of Real-World Data: Statistical Analysis Plan for the Optimise:MS Multicenter Prospective Cohort Pharmacovigilance Study
Source: Front Neurol. 2022 Mar 28;13:799531. doi: 10.3389/fneur.2022.799531 (PMC8996123; doi:10.3389/fneur.2022.799531)
Supplement: Supplementary file 1 [file Data_Sheet_1.DOCX]

Supplementary Material

# Inverse probability of treatment weights (marginal structural model)

Follow-up is divided into timeperiods of uniform length. Within each timeperiod, covariates and treatment exposures are held fixed at the latest available values at any time before the start of the timeperiod; values within the timeperiod itself may not be used. It is anticipated that timeperiods of 6 months will be used but this may changed if considered appropriate in order to achieve positivity and/or capture more frequent changes in covariates/exposures.

The weight $w_{\mathrm{it}}$ for subject $i$ and timeperiod $j$ is calculated as the product of a treatment selection weight $w_{\mathrm{it}}^{A}$and a censoring weight $w_{\mathrm{it}}^{U}$.

$$w_{\mathrm{it}}={w_{\mathrm{it}}^{A}\times w}_{\mathrm{it}}^{U}$$

$$w_{\mathrm{it}}^{A}=\prod_{r=1}^{t} \frac{P\left( A_{ir}=a_{ir}|\bar{A}_{ir-1},B_{i},C_{i1} \right)}{P\left( A_{ir}=a_{ir}|\bar{A}_{ir-1},B_{i},\bar{C}_{ir-1} \right)}$$

$$w_{\mathrm{it}}^{U}=\prod_{r=1}^{t} \frac{P\left( U_{ir}=0 |\bar{A}_{ir},B_{i},C_{i1},\bar{U}_{ir-1}=0 \right)}{P\left( U_{ir}=0 |\bar{A}_{ir},B_{i},\bar{C}_{ir},\bar{U}_{ir-1}=0 \right)}$$

where:

- $B_{i}$ is the subject’s baseline covariates
- $A_{it}$ and $C_{it}$ are the treatment exposure and time-varying covariates in timeperiod $t$ respectively
- $U_{it}$ is a binary variable with $U_{it}=1$ if subject $i$ is censored by timeperiod $t$ and $U_{it}=0$ otherwise
- $\bar{A}_{ir},\bar{C}_{ir}$ and $\bar{U}_{ir}$represent the history up to and including timeperiod $r$ of the processes $A_{it},C_{it}$ and $U_{it}$

In the definitions of $w_{\mathrm{it}}^{A}$ and $w_{\mathrm{it}}^{U}$ , the denominator of each term within the cumulative product is the probability of treatment or censoring respectively during timeperiod$t$, given exposure, censoring and covariate histories up until that point. The product of these terms therefore represents the probability of observing the entire treatment or censoring history up until that timepoint, under the assumption that treatment exposure and censoring in a given timeperiod $t$ depend only on the most recent covariate and exposure values (i.e. those from the previous timeperiod $t-1$). The numerators are similar but are based on only the baseline covariate values for each subjects; this helps to “stabilise” the weights in the sense that the weight distribution is more tightly centred around 1. Consequently the weights only control for the time-varying component of confounding and not any confounding that is present at baseline. In other words, because the probabilities in the numerators involve the baseline covariates $B_{i},C_{i1}$, the treatment effects are conditional on these covariates, and they must be included in the regression of outcomes.

The treatment and censoring probabilities (conditional on covariates and exposure from the previous timeperiod) will be fitted using pooled logistic regression models across all timeperiods. For some analyses when censoring is not expected to be substantial, the censoring model may be omitted, i.e. $w_{\mathrm{it}}^{U} = 1$ at all timepoints.

One disadvantage of the IPTW approach occurs when some observations have particularly low probabilities of their observed treatment, and hence particularly large weights. This gives these observations (which are by their nature outliers in the population) excessively large influence on the results and increases the variance of the treatment effect estimates. To account for this possibility, sensitivity analyses will be performed in which observations with extreme weights are “trimmed”, i.e. removed from the analysis. Two definitions of extreme weight will be used: (i) below the 1^st^ and above the 99^th^ percentile, and (ii) below the 5^th^ and above the 95^th^ percentile.

Weights will be normalised (after trimming, where applicable) so that the average weight at each timepoint is 1. Normalised weights $w_{it}^{N}$ are calculated as

$$w_{it}^{N}= \frac{w_{\mathrm{it}}\sum_{i\epsilon R_{t}} 1}{\sum_{i\epsilon R_{t}} w_{\mathrm{it}}}$$

Where $R_{t}$ is the set of subjects who are uncensored by timeperiod $t$.

# Weighted 2x2 contingency tables

For a given treatment and adverse event of interest, the disproportionality-based signal detection methods are based on the following count variables (which form a 2x2 contingency table of event versus treatment):

$n_{00}$ = number of patient-months without exposure to drug or occurrence of event

$n_{01}$ = number of patient-months without exposure to drug but with occurrence of event

$n_{10}$ = number of patient-months with exposure to drug but without occurrence of event

$n_{11}$ = number of patient-months with exposure to drug and occurrence of event

By applying the IPTW weights to the observed event indicators $Y_{ij}$ and non-event indicators ($1-Y_{ij}$) occurring on and off treatment (indicated by $X_{ij}$ and ${1-X}_{ij}$ respectively) within each patient-month *j* for subject *i*, weighted equivalents of $n_{01}$, $n_{11}$, $n_{00}$ and $n_{10}$ can be defined as

$$n_{01}^{*}= \sum_{i,j} w_{it_{j}}^{N}{{(1-X}_{ij})Y}_{ij}$$

$$n_{11}^{*}= \sum_{i,j} w_{it_{j}}^{N}{X_{ij}Y}_{ij}$$

$$n_{00}^{*}= \sum_{i,j} w_{it_{j}}^{N}{{(1-X}_{ij})(1-Y}_{ij})$$

$$n_{10}^{*}= \sum_{i,j} w_{it_{j}}^{N}{X_{ij}(1-Y}_{ij})$$

where $w_{it_{j}}^{N}$ is the normalised weight for the timeperiod $t_{j}$ covering month *j.*

Using these weighted counts, the total unexposed follow-up time is given by

$$n_{0.}^{*}=n_{00}^{*}+n_{01}^{*}=\sum_{i,j} w_{it_{j}}^{N}(1-X_{ij})$$

i.e. a weighted sum of the unexposed patient-months. Since $n_{01}^{*}$ is the weighted sum of event counts in unexposed patient-months, $n_{01}^{*}/n_{0.}^{*}$ is interpreted as the weighted estimate of the incidence rate in unexposed periods.

Similarly $n_{11}^{*}/n_{1.}^{*}$ is the weighted estimate of the incidence rate in exposed periods, and therefore the weighted proportional reporting ratio

$$PRR=\frac{n_{11}^{*}n_{0.}^{*}}{n_{01}^{*}n_{1.}^{*}}$$

is equal to a weighted estimate of the IRR. An equivalent estimate can be obtained by performing Poisson regression in a weighted pseudo-population in which there are $w_{it_{j}}^{N}$ copies of observation *(i,j)*. It can easily be seen that the entries in the 2x2 contingency table for such a weighted pseudo-population are equal to $n_{00}^{*},n_{01}^{*},n_{10}^{*}$ and $n_{11}^{*}$.

# Time-weighted cumulative exposure

Time-weighted cumulative exposure assigns more weight to recent exposures than historic exposures, allowing for a gradual “washout” of treatment effects. Different washout rates will be explored. For each subject $i$ and treatment category $k$, the time-weighted cumulative exposure variable at month $j$ is $S_{ijk}$, defined as

$$S_{ijk}=\sum_{m<j} e^{w(m-j)}X_{imk}$$

where $X_{imk}$ is a binary variable indicating whether the patient is exposed to treatment $k$ in month $j$ and $w$ is a parameter representing the monthly washout rate, i.e. the proportion of treatment potency that is washed out within a month. The estimated coefficient for $S_{ijk}$ quantifies the immediate effect of each month of treatment, which is then assumed to decay at rate $w$. Initially $w$ will be taken to be 0.5, i.e. a half-life of one month, meaning that each dose of treatment is 95% washed out after 6 months. Other values of $w$ may be explored.

# Example: longitudinal vs spontaneous report data in disproportionality analysis

Consider a fictional drug X and adverse event Y. Suppose the following exposure and incident event counts are taken from a longitudinal cohort study.

|  | Drug x | Other drugs |
| --- | --- | --- |
| Patient-months of exposure | 2 400 | 96 000 |
| Total events | 40 | 2 250 |
| Event y | 32 | 1 380 |

The incidence rate ratio (IRR) for event Y on drug X compared to other drugs is 32*96000/(2400*1380) = 0.93 < 1, telling us that event Y occurs less frequently on drug X than on other drugs.

The longitudinal formulation of the proportional reporting ratio (PRR) is equal to the IRR. The longitudinal formulation of the reporting odds ratio (ROR) is 32*(96000-1380)/((2400-32)*1380) = 0.93 < 1. The use of either of these disproportionality measures therefore will not trigger a signal, as expected given the lower incidence rate on Drug X.

The spontaneous-report formulation of the PRR is 32*2250/(1380*40) = 1.30 and its lower 95% confidence bound is 1.11 > 1. The spontaneous report formulation of the ROR is 32*(2250-1380)/((40-32)*1380) = 2.52 and its lower 95% confidence bound is 1.16 > 1. Therefore either of these disproportionality measures will trigger a signal associating Drug X with an elevated risk of event Y, despite the lower incidence rate on drug X compared to other drugs.

Similar results can be observed using alternative disproportionality measures.
